# Towards an effective transnational regulation of AI

**DOI:** 10.1007/s00146-021-01310-0

**Published:** 2021-11-09

**Authors:** Daniel J. Gervais

**Affiliations:** Vanderbilt University Law School, Nashville, TN USA

**Keywords:** Natural law, Ethics, Regulation, Agency, Transnational, World Trade Organization, United Nations, Kill switch

## Abstract

Law and the legal system through which law is effected are very powerful, yet the power of the law has always been limited by the laws of nature, upon which the law has now direct grip. Human law now faces an unprecedented challenge, the emergence of a second limit on its grip, a new “species” of intelligent agents (AI machines) that can perform cognitive tasks that until recently only humans could. What happens, as a matter of law, when another species interacts with us, can be integrated into human minds and bodies, makes “real-world” decisions—not through human proxies, but directly—and does all this “intelligently”, with what one could call autonomous agency or even a “mind” of its own? The article starts from the clear premise that control cannot be exercised directly on AI machines through human law. That control can only be effected through laws that apply to humans. This has several regulatory implications. The article’s first discusses what, in any attempt to regulate AI machines, the law can achieve. Having identified what the law can do, the article then canvases what the law should aim to achieve overall. The article encapsulate its analysis in a list of both doctrinal and normative principles that should underpin any regulation aimed at AI machines. Finally, the article compares three transnational options to implement the proposed regulatory approach.

“If we were to imprison the robot ﻿for nonpayment, why would it care?”(Russell 2019, 126).

## Introduction

The law is very powerful. It can strip someone of assets, order lengthy imprisonment or even death. As humans, the legal system can exert a tight grip on our behavior when we stray beyond the bounds of the law. Yet, as powerful as it is, the legal system can neither prevent hurricanes, nor force a crow to fly west instead of east.

The law contains rules according to which, “*human beings* are required to do or abstain from certain actions, whether they wish to or not” (Hart 1994, 81, 91). Human laws, in other words, are not meant for all agents, only for humans. More specifically, one could say that, because human behavior is thought to be controlled by the mind,^1^ the actual target of laws meant to regulate behavior (*ex ante* by imposing rules on behavior or *ex post* by dealing with the consequences of trespasses) is the *human mind* (Sapolsky 2018, 172).

Humans have occasionally invited into their legal order nonhumans such as animals, rivers and lakes, and ethereal entities created by humans and called “legal persons”(McGraw 2019; Rowe 2019).^2^ Yet, neither animals nor lakes have a mind that makes them responsible for their behavior in the eyes of the law. Legal persons operate as fictions, but “their” acts are in fact decided by human minds.^3^ Hence, those nonhuman legal subjects necessarily instantiate their rights through human proxies; “they are simply a vehicle for addressing human interests and obligations” (Cupp 2018, 591–592). The reason is self-evident: human laws can only directly affect human behavior. Simply put, “law” is a human invention. (Harari 2015, 28; McKinley Brennan 2002). Humans *make* the laws and the institutions that enforce them. Then human laws are written using language, another uniquely human invention (Chen 1995, 1278). I

As a result of the accelerated development of the affordances of AI machines, public bodies making (human) laws now face a unique challenge: regulating AI machines that can perform cognitive tasks that until recently only humans could—indeed machines can now outperform us at many of them.^4^ This challenge is unprecedented. What happens, as a matter of law, when another “species” interacts with us, can be integrated into human minds and bodies, actually makes “real-world” decisions—not through human proxies, but directly—and does all this “intelligently”, with what one could call autonomous agency or even a “mind” of its own?^5^ The question boils down to this: does human law have any grip on AI machines?

In 1993, Vernor Vinge predicted that a new form of AI he dubbed “superintelligence” would emerge and mean the end of the human era, and that it would likely happen before 2030. What he foresaw was a “singularity” that would take the form of an “exponential runaway *beyond any hope of control*” (Vinge 1993). This risk is very much alive today. There is a real risk that AI technology will ‘slip beyond our [human] control’ (Wallach 2015).Control of AI is thus a real issue, and the question that this article examines is whether and how *human laws* can achieve it.

This article explains and then starts from the premise that, because law cannot exercise control *directly* on AI machines, control must necessarily be effected through *laws that apply to humans,* especially those making and using AI code and deploying AI machines. If an AI machine is “law-abiding”, it will be because a human understanding of applicable rules and norms—or perhaps someday a method to understand those rules and norms—was embedded in its code (Chinen 2019, 147–149). This has, as the article explicates, a number of regulatory implications.

To set the frame of reference before embarking on our analytical journey, the article makes four liminary observations woven through the fabric of the article that will reappear in greater detail below. First, as just noted, it is beyond cavil that the law’s actual target is human behavior.Indeed, this is implicitly or explicitly what informs the design of regulatory targets (Klass 2012, 452). Second, as the law stands now, AI machines are not legal persons, which implies *doctrinally* that liability and ownership pathways can only target a human (or legal person).^6^ This means that even if AI machines can behave *like* humans—and in some cases much faster and better than any human can—we cannot regulate them exactly *as* humans or persons (Arnold and Scheutz 2018, 60; Wallach and Allen 2009, 109–110). Third, the use of AI by humans changes our behavior and, as our behavior changes, so does the law’s target, namely the human mind. For example, if harm caused by a human involves reliance on a decision made by an AI machine, it may be necessary to explore new causation pathways (Abbott 2020). This means that AI machines are changing the behavior of their would-be “regulators”. Fourth and finally, AI machines can *make autonomous decisions* (or something that looks like human decisions in terms of their effects^7^) that can have real-world impacts, whether by creating value (e.g., inventing something that could be patentable) or loss (a self-driving vehicle causing injury or damage^8^) (Yanisky Ravid and Liu 2018; Boeglin 2015).

The article explains sequentially what the law *can* achieve in regulating AI machines, what it *should* aim for overall, and then suggest possible ways to implement the proposed regulatory approach. The article’s first objective is to discuss what, in any attempt to regulate AI machines, the law can actually achieve. Answering that question is a necessary step before one can formulate *operational* regulatory proposals. Like an architect, we can plan new norms to regulate AI, but we need legal engineers to ensure that our house of norms is not one made of cards. The second objective is to identify what the law should achieve. The proposed solution is transnational in nature. The article also reviews possible appropriate institutional framework to implement the proposed solution.

Specifically, the article proceeds as follows. Part II describes and explains the new context in which regulators operate, namely their unprecedented task to regulate autonomous, “intelligent” nonhuman agents. This Part establishes the boundaries of what the law *can* achieve. The following Part focuses on a central, recurring theme in the literature about AI regulation and a key tool that the law can use to regulate AI machines, namely “kill switches”, which can be defined for now as hardware or software-based mechanisms to interrupt an AI machine’s processes, either temporarily or permanently. The article explains why those switches are necessary but far from sufficient. Then Part IV builds on the findings of the previous two Parts. It provides a detailed map for what the law *should* do to regulate AI machines beyond the use of kill switches. The Part argues that ethical norms must be programmed into AI machines, which means imposing behavior rules on human programmers, users and/or owners of AI machines. The article then offers a list of normative principles that should underpin any regulation aimed at AI machines to effectuate the proposed approach. It also illustrates their application using liability law. The article’s last Part proposes possible appropriate frameworks to implement the suggested approach and discusses and compares three transnational options to implement it.

One issue related to the article’s analysis is a discussion as to when humans should and should not be held responsible for the acts of AI machines when those machines are acting with a degree of operational autonomy, thus potentially breaking the causal link often required in tort law to impose liability on humans who own, program and/or use the machine. There is already an abundant literature on this topic (Abbott 2020; Kowert 2017). The article limits itself to identifying the borders of human liability in cases where this is necessary.

## The new context: nonhuman intelligent agents

### AI decisions

One of the salient features of AI machines is that they can make—and in some cases directly implement—a wide array of what one might legitimately call autonomous *decisions*^9^ (Etzioni and Etzioni 2016; Scherer 2018, 263).^.^ The High-Level Group on AI set up in June 2018 by the European Commission made this ability an element of their “updated” *definition* of an AI machine, which it now defines as a machine “designed by humans that, given a complex goal, act[s] in the physical or digital dimension … [and decides] *the best action(s) to take* to achieve the given goal” (Independent High-Level Expert Group on Artificial Intelligence 2019). Current examples of decisions made and implemented by AI machines include someone’s creditworthiness, or what advertisements or next video to send to individual users on Facebook or YouTube (UK Information Commissioner’s Office and Alan Turing Institute 2020; Solsman 2018; Pearce 2019; Coglianese and Lehr 2017). The US federal government is piloting an AI machine to make at least the initial call on entitlements to social security benefits (Engstrom et al. 2020). AI machines “are making decisions at higher and higher levels of authority in many areas. Take airlines, for example [machines] are taking over the job of managing disruption: rerouting planes, rescheduling staff, rebooking passengers, and revising maintenance schedules” (Russell 2019, at 130). AI machines make war and combat-related “decisions” (Chandler 2020). They are capable of selecting military targets and delivering force *without any human input* or interaction (Guiora 2017; Crootof 2016). The last example of an AI machine making life or death decisions while humans remain out of the decision-making loop is rare as technology stands now, but the degree to which a machine may act autonomously will vary, and in the case of military hardware the degree of autonomy is the result of human decisions (Wallach 2015, 337–340; Chesterman 2021, 104–105).

There are much more mundane examples. Consider that AI machines are driving autonomous vehicles in many U.S. states (National Conference of State Legislatures 2020) and elsewhere around the world. A car, whether operated by human driver, an AI machine, or a mix of both, constantly makes *decisions* (or something like it) about the operation of the vehicle. Those decisions can obviously cause harm (Wakabayahi 2018). The law already recognizes that those machines make the same kind of decisions (in terms of their effects at least) as humans.^10^

As a legal matter, the knowledge that putting thousands of autonomous vehicles on the road will necessarily lead to accidents, including injury and damage to property (although probably far fewer than vehicles operated by humans, possibly by two orders of magnitude), is the source of a challenge that the law must be able to meet (Lemann 2019, 172–175). One reason that explains why it is not easy to find the optimal standard to program AI machines used to drive on our roads is because driving decisions, whether made by human or machine, often have *moral implications* (Cataleta 2020; Gamez et al. 2020). For example, should an autonomous vehicle decide to swerve off a cliff and kill its passengers to save a school bus full of children? (Lin 2017; Naughton 2015; Polacek and Greene 2018; Thomson 1985; Tronsor 2018, 26–230).^.^ This variation on the well-known theme of the runaway trolley is “a vivid and apt way to capture a concern that future adopters of driverless cars will likely have on their minds: How will this car decide whether to prioritize saving driver and passengers or other people at the occupants’ expense?” (Huang 2019, at 1817–1818). It illustrates the hard work ahead and encapsulates the debates about the possibility of developing artificial moral agents (Formosa and Ryan 2020).

As the above examples demonstrate, AI machines definitely “act upon the world” (Calo 2015, at 529). And they can do so without relying “on moment-to-moment close control by a human”, as terms like “unsupervised machine learning” and “deep learning” suggest (Greely 2018, 2331). Naturally, AI machines sometimes only provide human decision-makers with suggestions but research shows that humans tend to follow those suggestions (O’Brien and Kang 2018, at 369–370; Nutter 2019, at 931–932). Despite the complexity of the picture that emerges, the common denominator to all the above examples is the autonomous and arguably intelligent *decision-making* ability of certain AI machines, decisions that impact human lives and property (Binns 2018). Though statutes in specific areas have already been adopted and more are likely to emerge, lawsuits to prevent, challenge or sue for the consequences of a decision (or omission/failure to decide) attributable to an AI machine will be one of the main tools available to build the interface between AI and the law (National Conference of State Legislatures 2020).

### AI risks

This section identifies two principal *categories* of risks associated with the types of decisions made by AI machines described in the previous section (Walz and Firth-Butterfield 2019, 186–197; Scherer 2016, 357). Risks of the first category arise when an AI machine is *specifically programmed by humans* to do something with negative and even potentially devastating consequences, like interfere with an election using an army of bots posing as Facebook users or disabling a country’s power grid (Stein 2020, 30–32). Naturally, the perceived valence of a specific act or decision and its consequences will depend on one’s evaluative framework.^11^ It seems more likely than not that human intent to cause harm using an AI machine will be laid at the feet of the human(s) who intended the harm even if the machines made some of the decisions implementing the objective, because both the end and part of the means were decided by one or more humans. One could also place in this first category injuries caused by an AI machine due to programming mistakes (glitches), as those errors are generally attributable to human programmers.^12^

The second category of risks comprises harm caused by an AI machine making autonomous decisions. In a typical scenario belonging in this category, the end might be decided by humans but the means are largely decided by the machine, based on its objective. The machine may well have been programmed to do something that is ultimately beneficial but what if it autonomously decides to use a destructive method to achieve its goal, with possible direct and collateral damage? Take for example a self-driving vehicle choosing which way to go in the case of an unavoidable accident (say, brake failure) but given no specific instructions as to how to prioritize potential harms. To simplify, assume that the self-driving car (AI machine) must choose between a similar number of potential victims no matter what decision it makes (say, go right, left or continue straight), in which case a quantitative formula would not yield a preference. Which other factors should it take into consideration? Should it factor in “qualitative” factors about potential victims? Should it decide, for example, not to prioritize saving a motorcyclist’s life because she is not wearing a helmet?^13^ Should it choose to save the driver (and perhaps owner) over the pedestrian, the old person over the young, or the other way around? It is not (just) a matter of ethics vs math. In the case of the motorcyclist, for example, the lack of helmet protection might actually increase the probability of death. This realization that autonomous vehicles make choices that are at “ethical” in nature has generated a debate over whether the ethical guidelines of the vehicles decision-making should be determined by the owner or operator (Lin 2014; Millar 2014).

The *autonomous decision-making* ability of some AI machines may break the causal link between human actors and outcomes—“machines without principals” (Vladeck 2014, at 146–150). At that point, the law may not be able fairly to impose liability on a human programmer, user or owner (Pagallo 2013). More importantly, the question to which one must return once again is, what *can* the law do to prevent harms of the second category? One of the most common ideas in the literature (both legal and fiction) about AI machines that misbehave is that we can always rely on “kill switches”—at least to stop ongoing harms and prevent future ones. This idea is explored in the next Part. Before doing so, the article explains the role of cognition in regulatory tools—including kill switches—used to target AI machines.

### AI, cognition and the law

AI machines can understand and apply the rules of chess, Go and poker if those rules are embedded in their code or learned by trial and error (Lien 2016). In fact, AI machines can beat the best human masters at all three games (Metz 2016).^14^ AI machines can also understand the rules of, and then beat the very best humans at, other games that require a high degree of what one might call creative thinking, such as StarCraft and Dota2 (Simonite 2017; Russell 2019, 56). Does the AI machines’ ability to follow those rules mean that those machines can understand (and follow) human law?

Human norms, in contradistinction to those that apply to even the most complex games, can be fuzzy or implicit; written or nonwritten; formulated or inferential; or, to use linguistic terminology, discursive or intuitive.^15^ Inferential (or non-formulated) norms play a major role in our behavior, effecting self-constraints that can only be understood by looking at “the sociocultural plane” (Castel 2014, 302; Farnsworth 2018, 1802–1803). Compliance with such norms “is accomplished, not by external constraint or threats of violence but through the interiorization of forms of self-control” (Castel 2014). This interiority is not “naturally occurring” but rather something that is shaped socially, carved into the individual psyche. This view finds an echo in work published by thinkers as diverse as Adam Smith and Immanuel Kant, who both emphasized the role of internal “moral constraints” in behavioral decisions (Stringham 2011, 99; Tegmark 2017). The Rule of Law itself is not “objective”; it is “deeply embedded in the rule of particular men and women” (Kahn 1997, 26–27).

Take a real human legal norm: the “reasonable person” standard. It is, of course, a fiction, for this ideal reasonable person no more exists than women who give birth to 1.7 children.^16^ How would that standard—or broader notions like right and wrong, or of good and bad behavior—be explained to an AI machine? (Gamez et al. 2020, 797). An AI machine could in theory be tasked with looking at a broad data set (say, all negligence-based tort cases since 1980) to infer reasonable behavior standards, using a so-called bottom-up learning approach (Walz and Firth-Butterfield 2019, 199). A jury, in contrast, cannot process that much data (Witmer-Rich 2018, 421).^17^ Which determination is better? A jury is likely to think in highly contextual terms in its consideration of notions like reasonableness (Chagal-Feferkorn 2018, 115). Jurors would likely factor in life experience, values and other similar considerations (Marder 2002, 666). This points to a key difference between human and machine: we can both do something one can call “thinking” about applicable rules and norms, but we do so differently (Bambauer 2017).

To illustrate the difference in our respective forms of thinking about the law and applicable norms, a human may be expected to understand a notion such as “common sense”, and a jury of her peers might take a defendant to task on it, but an AI machine? (Levesque 2017, 6 and 127; Wilson 1896, 232; Witherspoon 1955, 318). While AI machines’ decisions tend to be probabilistic and path-dependent, humans actually *expect* the law to jettison—or at least play down—the importance of what one might call “statistical perfection” to keep “the exercise of power intelligible and ensure that arenas like law enforcement, riven as they are with value-pluralism, maintain some measure of balance” (Brennan-Marquez 2017, 1300). How can that be explained to an AI machine? To answer that question, a focus on differences between human and machine cognition is useful—indeed it is necessary.

A veritable epistemological revolution is underway, as humans now depend on AI machines for many of their cognitive tasks (Holland 2018, 93). Put differently, the cognitive processes of humans who are trying to regulate the machines are being changed by the very machines that AI regulation targets. The machines are providing much of the data we use to make decisions, including about regulation.^18^ Looking ahead—without veering into science-fiction—one can identify a number of predictable factors related to ongoing cognitive changes that are liable to profoundly affect the legal order. That is part of a broader array of changes as AI machines are increasingly used to locate, filter and in some cases even validate legal information (Ihde 1990, 73).

Machines are changing how we interface with the world, and the law. Take two simple examples. First, billions of humans now outsource a significant part of their memory and cognitive functions to smartphones and similar devices. Those devices do not simply take over part of our memory function; they also make decisions for us (Fuentes 2017). Second, people who started driving a car before GPSs were omnipresent can still drive in cities where they drove before using a GPS without assistance from that technology, but are much less able to do so elsewhere without GPS (Javadi et al. 2017). It is, to put it succinctly, through AI devices that humans now “create, modify, organize, and access information distributed across vast remote networks” (Holland 2018, 93).

Why does this change matter? It is trite to suggest that information is a key input in human behavior, but perhaps less trite to realize that this means AI machines are already partly in “control” of our behavior. As humans have access to the incredible data processing abilities of AI, more of what we do will be data-driven, and so will the work of legislators, judges and lawyers (Guthrie et al. 2007, 43). Will a magistrate trust an AI that reports, say, a 72% risk of recidivism in a bail hearing, or will she trust instead her experience (and “gut”) that this defendant can be expected not to commit another crime? (van der Kolk 2015, 93). Cognitive research suggests that people tend to trust AI-generated statistics and suggestions because they “seem” scientifically grounded, which is far from obvious when one looks “under the hood” (Araujo et al. 2020; Logg et al. 2018). These changes matter, for cognition is intimately related to how the legal system functions. As technology changes our cognitive processes, it changes the legal order.^19^ The question is, while the ship changes course, who is at the helm?

Closely related to cognition, *language*—to which we can add the formation and transmission of concepts—is a unique human ability among living things (Pinker 1994, 24; Chen 1995, 1278). This is also true, indeed perhaps even more so, in law. As Cunningham neatly put it, using language “lawyers are autonomous creators of meaning” (Cunningham 1989, 2472; Hosticka 1979)^.^ For example, a reference to a statutory text is, in itself, only an “argument” as lawyers can argue about the meaning of words, and the role of words not used in the text. Even with creative ambiguity factored in, the text is not “the law”. One first must check for other norms, including possibly superseding ones.^20^ Then one must obviously look at cases, especially in a common law environment.^21^ Without offering an inventory of the full panoply of legal interpretation issues (splits among federal appellate circuits in the US, opinions that distinguish others, and so on), it is easy to see why teaching “the law” to an AI machine is not an easy task. Moreover, even if achieved at a particular point in time, it would have to remain dynamic, as legislation is amended and new opinions issued. Perhaps one day there will be an AI legal machine that other AI machines can turn to when they need to “understand” human laws. Although in theory, software is *plastic* enough to reflect human laws, whether it actually can do so depends on whether *“*programmers can implement [the] system they can imagine and *describe [it] precisely*” (Grimmelman 2005, 1723). Some notions may simply be unencodable: how does one teach the application of fuzzy standards like “reasonable person” or “common sense” to a machine?^22^ More generally, how can a programmer input *any* inferential and implicit norms into AI code? (Boddington 2017, 100–101). The challenge for programmers is to provide the “scripts” that prescribe actions of AI machines that are both within the bounds of the law and perform their intended function efficiently (Verbeek 2006, 362). In part IV, the article provides pathways for programmers to do just that. Before doing so, it considers the role of kill switches, a central feature in the literature about the regulation of AI.

## The regulatory role of kill switches

### The necessity of kill switches

This Part considers a core difficulty in regulating AI that stems from the fact that “well-programmed” machines may pursue their objective at all costs. The risk is that “no matter how wrong it is; they will resist attempts to switch them off; and they will acquire any and all resources that contribute to achieving the objective” (Russell 2019, 172). This means that AI machines might cause harms of the second category (as defined above). Companies programming the machines will want them to be as efficient as possible, which may, without proper guardrails, be interpreted by the AI machine as an instruction to use any means available.^23^ Though there is broad agreement that regulation of AI should include rules mandating human control, agreeing on the details of what level and form that control should take and how such control must be encoded, including by private entities over their own systems, is likely to take time and might vary by area (autonomous vehicles vs. high frequency trading, for example) (Chesterman 2021, 175) The article suggests, however, that as a simple precautionary matter, any AI system should by law include a kill switch. This will not be easy either. The problem regulators will face is that the regulation and the machine’s program may thus be *working at cross-purposes*: the law may want to control the machines but the machines may try to avoid constraints that restrict their ability to achieve their objective.^24^

Add to this a factor adumbrated by the opening quote of the article: in AI regulation there is what John Danaher calls a “retribution gap” caused by the differences between human and machine cognition described in the previous Part and that puts the spotlight directly on a major limit of the (human) legal order (Danaher 2016, 299).That problem is, essentially, that as autonomous, intelligent agents increasingly share tasks and space with humans, they will cause harm but when they do so, they will not be appropriate *subjects* of the retributive blame that explains much of the grip that the law has on humans (and legal persons controlled by humans). Recall that the law *cannot* regulate AI machines directly any more than it can regulate mathematics or physics; it can only regulate the humans who are making, selling and using those tools (Martin 2017).

The retribution gap follows from the fact that the two main tools traditionally available to the human legal order as enforcement mechanisms against an agent who has been found civilly or criminally liable—namely financial awards (compensatory or punitive) and imprisonment—do not work directly against an AI machine/agent.^25^ This implies that the deterrence function that the possible imposition of those penalties is meant to fulfill for humans would be similarly ineffective, even if somehow the machine “understood” the intended deterrence function (Turner 2018, 361).

The “punishment” that seems more readily available is to temporarily or permanently deactivate the AI machine by using what is colloquially referred to as a “kill switch” (Turner 2018, 364–365). During the deactivated (“down”) phase, the machine may be “rehabilitated”, that is, its code can be debugged. The idea of a kill switch as the “ultimate form of regulation” has been used to generate drama in many a sci-fi creation: the kill switch as proof of human control over a runaway machine.^26^ Beyond sci-fi, the idea of a kill switch has made regular appearances in both the popular press and specialized AI literature (Margolin 2016).

#### The limits of kill switches

Kill switches *can* fail—in real life, not just in sci-fi novels. They can be poorly designed of course, but consider that the machine has a teleological interest in resisting because a kill switch is, for a machine trying to accomplish a task, the *ultimate cross purpose*.^27^ A “terminated” machine or process cannot or no longer accomplish its task (Turner 2019, 363). Oxford University’s Nick Bostrom has made two related, chilling suggestions in that context: first, that there could be an “existential catastrophe as the default outcome of an intelligence explosion”; and, second, that kill switches are unlikely to work because a machine might detect and disable its kill switch (Bostrom 2014). To avoid this catastrophic outcome, a number of proposals have been made to program *only* “Safely Interruptible AI Agents” (Orseau and Armstrong 2016). The underlying idea is to be able to “take control of a robot that is misbehaving and may lead to irreversible consequences”, by creating a “framework to allow a human operator to repeatedly safely interrupt [an AI agent] while making sure the agent will not learn to prevent or induce these interruptions”(ibid). To avoid the cross-purpose issue, the implementation of this framework requires that the machine “be uncertain about the utility associated” with the use of the kill switch, perhaps seeing its use as a way to get improvements (Hadfied-Menell et al. 2017, 221).

There are at least two problems to solve to implement mandatory, omnipresent, and effective kill switches.^28^ First, unlike “an individual human, who can only be killed once, [an AI machine] can exist in various iterations or copies. These might be distributed across a wide geographic network” (Turner 2019, 367). One simple way to minimize this risk is to make it mandatory *for human users* of at least mass-market AI machines to download and install updates, for example as a condition to maintain an insurance policy on an autonomous vehicle.

Another, less obvious potential difficulty is related to the changes in behavior in humans induced by our increasing reliance on AI machines. AI machines may “learn to manipulate humans so as to either activate or deactivate [the kill switch]” (Turner 2019, 364). To encapsulate in a few words a complex point, to a machine “we are all subject to the ebb and flow of the varied emotions that, to a large extent, govern our behavior” (Sellars 1997, 232). An advanced machine might realize that it can easily influence human thinking, just as, say, Facebook’s algorithmic choices about the information provided to users can sway election outcomes, as has now been demonstrated in several countries around the world.^29^

This is not sci-fi. In a 2019 interview, the co-founder of Applied Invention, self-described as a “group of experienced technologists that have especially strong skills in building complex systems that incorporate […] artificial intelligence [and] robotics”, explained that “AI bots are adept at evolving their behavior in ways that are very difficult for the designer to predict […] Sometimes those forms of behavior are actually kind of self-preserving, that can get a *bit out of sync with the intent and goals of the designer*”.^30^ Even for experts who dismiss outsized claims about existential threats posed by AI (and subsequent press treatments of superintelligence) as a combination of futurism and alarmism, “the challenge of evaluation and responsible implementation is an all-too-real struggle here and now” (Arnold and Scheutz 2018, 78).^31^

Kill switches are part of what the law can and should do. They are, in other words, part of the regulatory approach proposed by this article. Even if effective and universally implemented, however, kill switches do not provide a full answer to the question what the law can and should do to regulate AI. The next Part incorporates the article’s findings about kill switches but also charts a broader regulatory path forward.

## A comprehensive regulatory approach

Based on the discussion in the previous two Parts, this Part sets forth proposals to deal with the challenges to the legal order as humans and machines learn to cohabit. It considers, first, the tools that the law has at its disposal to regulate AI machines and their limitations. It then considers the challenges involved in programming the type of behavioral rules into AI machines that the legal order imposes on humans. The next step is the proposal of a comprehensive regulatory approach, consisting of two sets of principles, doctrinal and normative, that reflect the article’s findings. To illustrate the proposed approach’s application, the last section of this Part applies it to liability analyses.

### The limits of the legal order

As the previous Parts explained, enforcing human laws on or against AI machines will not be easy. To take a simple example, if a court were to issue an injunction to “order” a machine to do, or stop doing, something, that order would be useless *in se*.^32^ The machine’s “refusal” to obey an order would not be enforceable by imposing a monetary penalty (on the machine) or a prison sentence. The order’s force, if any, would depend entirely on humans responsible for the machine’s behavior and whether they are willing—and perhaps more importantly able—to obey the order.^33^ Someone “will have to translate that injunction, written in legalese, into code the robot can understand” (Lemley and Casey 2019, 1370). Yet, as Chesterman rightly notes, ‘the idea that relevant ethical principles can be reduced to a few dozen words, or that those words might be encoded in a manner interpretable by an AI system misconceives the nature of ethics and law. (Chesterman 2021, at 174). Programmers face significant constraints as coding the exact scope of the order may not be obvious for reasons alluded to above. The order may naturally involve the use of a kill switch to stop and then perhaps reprogram the machine.

Then, as noted in the previous Part, for an AI machine compliance with human law overlooks what may well be a direct conflict with the machine’s program and objective. As explained in the discussion of kill switches in the previous Part, an AI machine programmed to achieve a specific set of objectives “will certainly understand that it will fail in its objective if it is switched off before completing its mission” (Russell 2019, 138). The harm the machine can cause may not be something the machine can actually grasp. Think of “how content-selection algorithms on social media wrought havoc on society in the name of maximizing ad revenues” (ibid., 140). Consequently, it seems “very hard, and perhaps impossible, for mere humans to anticipate and rule out in advance all the disastrous ways the machine could choose to achieve a specified objective” (ibid).

Recall the article’s premise that the law’s actual target was, is, and remains, human minds. The most direct consequence is that reaching the law’s target relies on the existence of sufficient links between humans that can affect a machine’s behavior and those machines. Machines “understand” code—code is their “law”—and this means that the rules that they follow must be embedded in their code.^34^ As AI machines increasingly partake in various aspects of life, our legal order’s expectations that “everyone” will abide by (human) laws (or suffer consequences from noncompliance) actually apply only to humans, not machines. In sum, this means ensuring legal compliance in one of two ways: through code and kill switches.

### Proposed framework

Let us restate briefly three conclusions reached thus far. First, the actual “regulation” of AI machines will be achieved *not by (human) laws but by code*. Legislators are not in the business of coding, but they can make laws that target human programmers and users of AI machines.^35^ Whether human law is actually “obeyed” by the machine will depend on its code and instructions received. Second, kill switches are useful and may well be necessary, but they are insufficient and may not always work. Even when they do work, they are no panacea and will certainly not be able to remedy harm already caused (Scherer 2016, 388). Simply put, the solution to any and all harms associated with the introduction in our midst of a new category of intelligent agents cannot always be to “pull the cord”. Third, an AI machine’s ability to find, interpret and behave according to the entire body of “human law” in the same way as humans is not likely something that AI machines are likely to be able to do anytime soon.

Based on those three findings, the article can now suggest a regulatory approach. This approach is based on what the law can do: ensure that AI machines are programmed to ensure that they will abide by a set of principles represented explicitly in AI system design and decision-making algorithms (Arnold and Scheutz 2018). Implementing this approach means both “telling” machines (using code) to weigh possible ways to perform a task according to their consequences and giving the machines factors to weigh in addition to efficiency (Siebecker 2019, 148).^36^ For example, Facebook’s AI bots are reportedly tasked with placing politically flavored posts in users’ feeds to maximize polarization, which has been said to harm democracy and should be regulated (Zittrain 2014; Edelman 2019; Weintraub and Valdivia 2020)^.^ This has led to specific regulatory suggestions to force Facebook’s programmers to modify their code (Grafanaki 2017, 58). As this simple yet important example shows, the proposed regulatory approach *can* be enforced through human laws because the law can impose obligations on companies, programmers and users (Berman 2018, 1330; Sloan and Warner 2019). Regulations can include more specific steps such as mandating the use of reinforcement learning to prevent AI machines “from learning the wrong thing… either by a person or the environment” (Arnold and Scheutz 2018, 62).^37^

A code of ethics is one of the best pathways to implement the proposed approach.^38^ The machines’ code (software) can embed *that* code (ethics). This naturally presupposes that one can determine a proper set of ethical rules—a controversial topic, especially on a planetary scale.^39^ Instead of proposing a single code or set of ethical guidelines, which it sees as suboptimal for reasons discussed below, the article will suggest appropriate processes for the development of such norms.

However they are developed, with time coded ethical rules or guidelines will become more standardized and the de facto “law” of many AI machines. Hence, independently of the approach chosen to define the proper set of ethical rules, this much is clear: whether an AI machine will follow (human) law *depends on the code*, and *code depends on humans* whose behavior human laws can control. This view is what informed, for example, the extant imposition on programmers of rules concerning the design of AI machines that can “explain” their decisions, so-called “white box” systems allowing ex post assessment of the machine’s operation and adherence to ethical norms (Deeks 2019; Doshi-Velez and Kortz 2017). Embedding the proposed approach through ethical rules in AI code is a powerful regulatory approach not only because that code is the AI machine’s “law”, but also because humans themselves must often “abide by” that code.

Even the best code of ethics may not be sufficient, however. AI machines are likely (like humans in that respect) to make many errors during their learning process as their decisions will be influenced by socio-economic and other conditions reflected in the dataset they are working from.^40^ One can thus foresee the need for regulation to prevent humans from using those systems in their early learning phase in a context where they can do harm—that is, until they have reached a level of safety.^41^

In appropriate cases, the law should also require, in addition to embedding ethical rules and rules preventing the use of systems before they are ready, public access to code/algorithms or their assessment on a confidential basis by a third party based on a set of regulatory guideposts (Bloch-Wehba 2020; Hern 2020). Such “transparency” regulations make it easier for humans to see how other humans have implemented their obligation to embed ethics into AI code (Robbins 2020). This might explain why such obligations have “intuitive appeal” (Selbst and Barocas 2018; de Fine Licht and de Fine Licht 2020). The same can be said of regulation requiring a “human decision”, or human review of a decision made by an AI machine that caused harm, which does not imply that a human will make a better decision, only that the law will have an identifiable legal target because the decision has caused harm (Huq 2020).^42^

### Application to liability

There is an abundant literature on the liability of AI machines (eg Pagallo 2013; Abbott 2020; Vladeck 2014; Rosenberg 2017). It is not within the article’s purview to offer a full discussion of this issue. To illustrate the utility of the proposed approach, the article can, however, briefly outline how it would apply to the liability for harm caused by AI machines.

Applying the proposed approach in that area of law would lead to three conclusions. First, both because the machine cannot be expected to understand and apply human law, and because the law has no direct grip on the machine due to the retribution gap and the other factors identified above, the target of any liability inquiry will be humans (directly or as agents of a legal person). Those humans will be, inter alia, programmers and users of AI machines. Second, the ubiquity of AI machines means that in some cases the human or legal person who might be liable will not be easily identified or will be in an out-of-reach jurisdiction.^43^ Third, bearing in mind proximate cause is at root a normative determination, it is at least conceivable in some tort cases that the law may decide, based on a proximacy analysis, not to impose liability on any natural or legal person, thus leaving no effective remedy to compensate the victim.^44^ An elegant solution fully explored elsewhere to remedy those situations and potentially increase buy-in into AI generally would be the establishment of an insurance system to compensate victims of harms caused by AI machines (Joshikawa 2019; Selbst 2020).

### Proposed principles

The proposed approach can now be translated into a set of five normative principles informed by the discussion in the previous pages.^45^

### Normative principles

(N1) All AI machines should be programmed with a kill switch to deactivate the machine temporarily or permanently. The kill switch code should prevent detection and deactivation of the switch by the machine. This should apply to all “copies” of the machine’s code;

(N2) Human-defined ethical behavior rules should be embedded in AI Code. This means that human programmers (or an associated legal person such as the employer) could and should be held liable, in appropriate cases, if and when it can be shown that they were negligent in not considering relevant ethical issues, which would be measured against ethical norms applicable in the programmer’s field of activity;

(N3) Ethical rules should apply to humans responsible for AI machine’s learning, including data sets that will be used to inform the machines’ decision-making process (Chopra and Singh 2018);

(N4) The use and/or degree of autonomy of AI in certain areas should be prohibited or severely limited where the risks clearly outweigh benefits by imposing serious penalties on humans who design, market or use those systems. This could include preventing or limiting use and access during the initial learning phase(s) (Shuklenk 2020; Wallach 2015, ch 12);

(N5) In cases where no person (human or legal) can fairly be held liable due to the absence of (proximate) cause, an insurance scheme mandated by law should be created to compensate for harms. Such a scheme could have a positive effect on the acceptance of the more widespread use of AI machines.

### Unpacking the principles

**Principle N1** is relatively straightforward. Kill switches were discussed in detail in Part III. According to principle N1, a kill switch should be mandatory in AI Code, even though kill switches are prospective tools and thus only perform part of the regulatory function. Applying the article’s analytical framework, enforcing a kill switch obligation using human law requires both an obligation imposed on human programmers to code kill switches into all AI Code and an ability for the legal system (that is, a rule) to find that one or more humans (directly or via legal persons) can be held liable for failing to program or use a kill switch if ordered to do so.

**Principles N2 and N3** require more unpacking. They revolve around the embedding of ethical rules into code. As noted earlier, this will not be easy, nor is it likely to be possible to embed all ethical rules into code (Chesterman 2021, at 174). One must thus answer the question “whose ethics?” To ameliorate outcomes, Pasquale’s suggestion that the rules of ethics should be decided democratically rather than by unaccountable AI firms seems unimpeachable (Pasquale 2020). But even if one accepts that as a high-level mobilizing idea, the difficulty of writing effective rules remains.

As the article sees it, embedding human-defined ethical rules in the code of AI machines is necessary to limit harms of the second category (as defined above), but it is not an easy task for at least three reasons. First, making a single set of planet-wide ethical rules for all AI machines is not, in this article’s view, achievable, nor indeed is it desirable, as a more pluralist approach is likely to be more appropriate (Elkus 2016). Second, it is fair to ask whether those codes of ethics will be enforceable at all. Third, in implementing principle N2 in particular, a difficulty that scholars have identified but failed to solve is the level of abstraction of ethical rules embedded in code, that is, whether to implement high-level standards and let the machine make mistakes, or apply ethical micro-directives in the code that leave the machine very little wiggle room but may not cover all situations.^46^

Dozens of proposed codes of ethics for AI already exist (Guihot 2017, 425). Despite this proliferation, however, “clear approaches and guidelines for data ethics in AI […] are still lacking”, due to two main factors: the complexity of agreeing on a proper set of ethical rules and finding ways to actually program them into the machine (d’Aquin et al. 2018, 54). After a review of several existing and propose codes, Chesterman concluded that the codes tend to coalesce around six ‘themes’: human control, transparency, safety, accountability, non-discrimination and privacy (Chesterman 2021, at 177–178). A rapprochement among various approaches is a positive development but one can readily see that an agreement on a potential list of areas of a code of ethics should cover is far from an agreement on the actual content of the rules to be enshrined in said code. This article does not propose a full code of ethics. Indeed, the article would go as far as to suggest that at this juncture at least, no one should. As alluded to already, a single code of AI ethics is likely not the optimal way forward. As Wallach and Marchant have suggested, the international governance of AI must remain *agile* if it is to succeed (Wallach and Marchant 2019). Societal conversations about sector-specific and national/regional codes or ethics boards should continue on this topic must continue.^47^ The article’s suggestion for the short term is that all AI Code should be written with ethics in mind, and that a programmer should be able to show that she considered a credible source of ethical rules. Then something like a reasonably ethical programmer standard might emerge. Programmers may be expected to use existing codes as checklists of issues (Wasilow and Thorpe 2019, 38). A longer term, more comprehensive solution is suggested below.

**Principle N4** reflects the fact that ethical rules have inherent limits. In some cases, the use of AI should simply be banned and humans held liable for transgressions. Certain other uses could be severely restricted, using a sliding scale of risk (Bathaee 2018, 936; van Asselt and Renn 2011, 436–438). This article suggests imposing such restrictions when AI use might “compromise human life and social stability” (Rhue and Washington 2020, 378). Although this imposition would prevent deployment of AI drones with the power to inflict (lethal) harm, for example, it can also be applied to less dramatic contexts such the reliance on AI for bail and sentencing decisions until those systems can be shown to be bias-free (Chesterman 2021, 33 and 61; Hillman 2019). Applying this article’s framework to any of those situations, undeniably the only legal recourse will be against humans. This means finding and being able to take legal action against humans who crossed the line by developing or using AI machines in a prohibited fashion.

As the conversation about AI ethics progresses, individual companies, trade associations and numerous other sources will continue to produce and update codes of ethics for AI (Bird et al. 2020; Luxton 2014; Wild-Raidt 2020; Boran 2018). Industry-based codes are likely to state only general principles that may not be easily enforceable, by using language requiring that the code should be “as transparent as possible” (which can be countered with a trade secret claim to limit disclosure) or that it should “not favor any type of users” (which is not to say that it *would* not).^48^ Government-issued codes should be more concrete and include at the very least both the kill switch and maximum transparency to allow humans to diagnose and fix any problems.^49^

What may matter more in the medium term than the substantive contents of ethical rules embedded in AI code, however, is the *process* by which the necessary conversation on AI ethics proceeds (Boddington 2017, 99). This is a societal conversation that must be had—indeed one that many different groups are having—but it requires, in this article’s submission, a more coordinated response. This work must be undertaken not by “private parties with commercial objectives, but by independent public bodies” (Kop 2020, 337).

Finally, **Principle N5** borrows a solution proposed elsewhere that can be used to remedy harms caused by AI machines that in all likelihood kill switches and ethical rules cannot fully prevent.

## Operationalizing the approach transnationally

Having proposed a regulatory approach and principles based on said approach, a major question remains, namely *how* the proposal can be implemented institutionally. This is the topic of the first section of this Part. The last Section offers broader perspectives on the future relationship between humans and machines.

This Part will argue that the role of independent public bodies mentioned at the end of the previous Part should be played on a transnational stage, for two principal reasons. First, because like anything operating in the digital environment, AI machines are active in more than one jurisdiction around the globe (Erdélyi and Goldsmith 2018; Westerheide 2019). Second, a country imposing a comparatively stricter regulation on its AI industry may put itself at a regulatory disadvantage (Martin 2017, at 106).

### Towards a transnational implementation of the proposed approach

This Part explores three possible transnational institutional paths to implement the proposed approach and normative Principles in an effective way. There are many others that could be mentioned here, including the G7, G20, or enforcement of soft law being developed by non-governmental organizations such as the IEEE.

The first proposed path would be for a group of like-minded countries to take the lead in developing the Principles—and in particular a code of machine ethics—and then project this approach around the world, possibly through trade and investment agreements.^50^ The second path is leadership by the World Trade Organization (WTO), which has done a significant amount of work on digital trade despite navigating in troubled political waters over the past few years (World Trade Organization 2020a, b). The third and final path would be an international treaty under the aegis of the United Nations or one of its specialized agencies. Let us explore the advantages and disadvantages of each path.

The first path is the quickest and could start from existing codes already proposed by governmental sources in, e.g., the European Union and the United States. We see a clear example of this approach in the December 2020 proposal for a Transatlantic Agreement on AI put forward by the president of the European Commission (European Commission 2020). Instead of just two major players, the problem could be addressed by a somewhat larger group of countries, as the example of the work done at the Organisation for Economic Co-operation and Development (OECD) demonstrates.^51^ Such an approach risks generating a “country club” approach to international negotiations, where a group of like-minded countries agree on a set of norms and then try to multilateralize them by getting other nations to “take it or leave it”, thus cutting the global conversation short (Gervais 2012a).

The second and third proposed paths, in contrast, are slower processes than the first but they can ensure broader participation, including by developing and least-developed nations that may not have the same interests as major AI-developing nations (World Trade Organization, undated; United Nations 2019). A fair critique of both approaches is the historical lack of effectiveness of intergovernmental organizations in responding to technological innovation (Chesterman 2021, at 204). That said, this is not a sufficient argument to reject the idea of even considering such options from the outset.

A broader framework could lead to the development of a norm-set that allows for a degree of pluralism necessary to let various jurisdictions perform “regulatory experiments”.^52^ Although the article takes the view that while embedding ethical rules in AI code is essential (Principle N2), defining a single set of top-down planetary norms is undesirable and indeed most likely impossible as a practical matter unless such norms are shrouded in a vagueness that would deny their effectiveness.

How do the WTO and the UN compare as possible vehicles to implement the proposed approach? The WTO can claim past successes, for example, in getting its diverse membership to agree on a single set of norms in the field of intellectual property, which was no small feat (Gervais 2012). The United Nations is obviously also more than able to negotiate new treaties, and it has already done some work on AI ethics (Azoulay 2019).^53^ An obvious difference between the two organizations is the normative underpinning of discussions under their respective aegis. At the WTO, the trade-focused approach tends to be anchored in economic considerations, most notably trade liberalization (Yakovleva 2020, at 510). Key differences emerge on other levels as well. The choice of forum (UN v WTO) changes *what* (trade-related at the WTO vs broader rules at the UN) is discussed, *how* it is discusse1d (the WTO has a less public process), and *who* is there to discuss (WTO Members self-evidently send mostly trade experts to negotiate there) (Howse 2002, at 98; Lim 2021).

Overall, as this article sees it the WTO’s ability to address AI regulation with a sufficient degree of purchase from various stakeholders will depend on the normativity of trade liberalization when measured against other societal goals, notably the development and deployment of ethical and humane AI machines. As things stand now, the normativity of trade bodies does not bode well for a successful outcome there.^54^ That said, the WTO has been under pressure to adopt a more pluralist stance and relativize the normativity of trade liberalization in the face of global public health crises and other planetary challenges—notably climate change (Gathii 2006). The WTO has arguably shown both an ability and increased willingness to consider the impact of its rules outside of the trade realm (Gervais 2021b).^55^ This may be even truer after the appointment of its new Director-General in early 2021.^56^ By comparison, leadership by the UN could take a more open-ended approach to goals and adopt a broader perspective that AI should be developed to be beneficial to humans.^57^

There will be predictable major opposition from industry against any mandatory set of rules. To take one small example, the EU has proposed an ethical rule according to which “[h]umans need to be aware that they are interacting with an AI machine” (European Union High-Level Expert Group On AI 2019a, b). One can doubt whether Facebook, to name just one stakeholder, would actively support such a recommendation, forcing it to identify the large number of posts made by AI machines (bots) not humans (Shao et al. 2018, at 5). As already noted, during any such multilateral process, one can expect soft law tools (especially prolapsed by industry) to play a prominent role, ideally as a steppingstone to binding international rules though perhaps they would be used as a way to forestall the development of such rules.^58^

There is another risk. Any code of ethics agreed upon by a group of nations will be cast in the stone of the document in which it is written and thus could not have the dynamicity required to adapt to changes in AI technology (Boddington 2017, 69–74). This risk should not be overestimated, however, as it can be managed. If one uses intellectual property as a possible parallel, patent law has barely changed in 200 years and, internationally, it is cast in the stone of the 1994 WTO Agreement on Trade-Related Aspects of Intellectual Property Rights or “TRIPS Agreement” (Gervais 2021a). This has not prevented the adaptation of patent standards to successive waves of transformation and evolution of the technological landscape (Pegram 1991, 19). This suggests that the way to mitigate the risk is to write norms in a technologically neutral fashion and interpret them dynamically—e.g., by national courts (Birnhack 2013, 38–39).

Finally, any plurilateral or multilateral treaty or decision must be implemented by each jurisdiction, adding delay but also a degree of flexibility allowing for pluralist “norm competition” (Patchel 2009; Krisch 2010, 78 and 103). To maximize the benefits of this competition, any transnational solution should be accompanied by an “observatory” function reporting on the implementation of the treaty or other set of agreed norms (World Trade Organization 2019).^59^ This function can shed light on implementation and may provide useful models for other countries to follow.

### An interspecific justice horizon

Potential interspecific justice issues involved in the cohabitation on Earth of two intelligent species are on the distant horizon—for now.^60^ As with ethics, there are several conceptions of “justice” that may come into play (Habermas 1996, 1499–1500).

The article’s has shone its analytical spotlight on the difficulties of integrating into daily life AI machines that make autonomous decisions and perform actions that can cause harm or injury.^61^ It is possible that, in the not-so-distant future, AI machines will be able to overcome the cognition problems discussed above and understand (in their own way) “our” legal order. Some see this as an ominous development—one which has filled much sci-fi literature—in which AI could become “self-aware machines taking over the world and destroying humanity in the process” (Krishnan 2009, 154). Some have labeled this emergent behavior a “singularity” (Kurzweil 2005)^.^. It is possible, to riff off Bostrom, that machine self-awareness will happen in a blink of an eye, be immediately out of human control, and mark the demise of our species. It is more likely, in this article’s view, that the first sputters of machine self-awareness will give humans sufficient warnings to react. It is equally likely that humans will not heed them*.*

Using a precautionary principle framework, this risk of emergence reinforces the role of kill switches because the level of harm could be catastrophic (Gervais 2010, 697; Chesterman 2021, 182–183). The harms caused by AI machines are more likely to be on a more mundane scale, however. This article prefers to be neither techno-utopian nor techno-dystopian, two viewpoints that differ radically on the correlation between technological progress and social progress (Yu-Xiao and Su-Tong 2018). The truth is probably in between: AI machines will do both good and bad. One of the most troubling and insidious harm is the replacement of humans at tasks that make human life meaningful, diminishing our capacity to perform demanding tasks—indeed our desire to do so—and generally “hollowing out human expertise” (Freeman Engstrom and Ho 2020, 854).

Looking further down the road, how will AI machines achieving higher levels of cognitive ability “deal with” humans, their “nonrational” behavior and our “fuzzy” legal order?^62^ Will they ask for rights under “our” legal system? Just as bots can be used to affect human elections, AI bots might be coded to, or even infer that, influencing policy debates on the regulation of AI is in their interest.

Finally, while it seems relatively straightforward to deny “human status” to AI machines, could they be “persons”? The question to ask, the article submits, is not whether the law *could* deem an AI machine a person. In theory, anything can be deemed a “person” by statute including a lake, if the legislator so decides, as explained in the Introduction. Not surprisingly, there are proposals to give that status to some AI machines (Bayern 2014, 1497). Some see such proposals as uncontroversial in the least because, after all, corporations and a number of other “creatures of the law” are “persons”, a statement which, as a matter of positive law, is certainly accurate. For corporations and similar entities, however, there may be an assumption that the moral agency of *humans in control* informs their decisions (Rothenberg 2016, 456–457).^63^ Whether this assumption will hold in the future is questionable (Lenk 2017). One way or the other, however, the same assumption does not apply to AI machines with independent agency, in the sense that there is often no direct and substantial causation between their action/decision and humans. Hence, this article does not support granting person status to machines for two main reasons. First, the fact that the law *can* give machines legal personality is not argument to support a claim that the law *should* do so. Second, until and unless machines actually understand and can be forced (like humans and legal persons) to abide by human rules directly, then it makes little sense to invite them to our table.

## Conclusion

AI machines make decisions. In some cases, those decisions are made autonomously, in the sense that there is no obvious way to attribute their cause to one or more specific human beings. The scope of this phenomenon is likely to increase over time as AI machines become more developed. Some decisions made by AI machines will cause harm to humans; others will generate benefits. Yet, AI machines cannot, as matters stand now and for the predictable future, be expected to understand and abide by the rules that govern human behavior. The human legal order is limited in that it can only target humans and legal persons—specifically programmers and users of AI machines. The legal order can and should use that ability to impose that programmers and users follow code kill switches and ethical rules into AI Code allowing AI machine to weigh possible courses of action against a set of human-defined values. The article has proposed an approach, and explained why and proposed possible modes of institutional implementation on a planetary scale.

## Footnotes

1 Or the brain, though this does not mean with (complete) free will. Indeed, “free will is threatened by engineered determinism. Free will is a person’s situated capability to reflect upon on and determine their beliefs, preferences, values, and intentions” (Frischmann and Selinger, 2018, 12–13).

2 It went a step further. Criminal trials of animals was not uncommon in medieval Europe (Chesterman, 2021, at 85).

3 Though increasingly also with the help of artificial intelligence machines (Siebecker, 2019; Gordon, 2020).

4 This article uses the term “machine” as a generic term that may apply to a computer using Artificial Intelligence (“AI”) software but could also cover machines capable of movement (i.e., robots). The term “systems” would be equally appropriate in this context. There is no universally agreed upon definition of “AI”. Whether the notion can be define ontologically or should be defined more functionally or phenomenologically is a matter of considerable debate. See eg Casey and Lemley (2020). The definition used by the European Commission is useful: “Artificial Intelligence (AI) refers to systems that display intelligent behaviour by analysing their environment and taking actions—with some degree of autonomy—to achieve specific goals” [emphasis added] (European Commission (2018)).

5 The meaning of the notion of “intelligence—the “I” of AI—is disputed. For example, if one means intelligence as measured by standard IQ tests, then AI machines are intelligent. This is easily explained. Those tests often use geometric progressions, Fibonacci sequences and similar tools, which are child play for a simple computer, even without AI capability. See Dowe and Hernández-Orallo (2012); Sanghi and Dowe (2003). For our purposes, one can define “intelligence” (the term “sapience” would likely be more accurate) as the ability to obtain information from one’s environment, turn it into knowledge and then use it to make decisions or take actions. Defined in this way, both humans and some AI machines are “intelligent”.

6 “Doctrinally” is used here to signal that this is not the only dimension. As noted above, legal subject status was previously given to entities that do not have a mind of their own. With AI, this may be different. Whether granting legal personhood to AI machine sis normatively desirable is another question. In line with this article’s analysis, the other question is how would the legal system interface with these persons and, where necessary, force them to obey (human) law.

7 See n 9 below.

8 Indeed, in the US state of Tennessee, the AI machine is considered the actual ‘driver” of an autonomous vehicle (Chesterman, 2021, 40).

9 Much of the literature in this area accepts the notion that machines make decisions, even if the decision-making process is not the same as for humans (See eg Chesterman 2021, 32–33; Gawdat 2021, 9).

10 For example, a Nevada statute provides that “[t]he automated driving system of a fully autonomous vehicle shall, when engaged, be deemed to fulfill any physical acts which would otherwise be required of a human driver except those acts which by their nature can have no application to such a system.” NRS 482A.200 (2019).

11 An example of that would be the deployment of Stuxnet, a “a computer worm that infected Iranian industrial sites, damaging its uranium enrichment stations and dealing a real setback to Iran's nuclear ambitions” (Kovach 2014, 34).

12 Also relevant for our purposes is the fact that some AI machines can write or enhance their own code (Metz 2020; Grothaus 2018).

13 Recall that AI machines can process much more data much faster than humans even in the fraction of a second that may be available for such a decision.

14 Someone might retort that those are not “real life” situations because they are games with generally straightforward and fully explainable rules. Poker is somewhat different than chess and Go, as it is an “incomplete information” game (Lien 2016).

15 The article borrows this terminology from (the late) Roderick Macdonald. On the distinction between intuitive v discursive norms, see McCauliff, 2009; Carmody Tilley, 2017, at 1326.

16 That is, the 2019 US fertility rate (World Bank, undated).

17 This arguably is what “common sense” is based on. Top-down means interpreting a rule or arguably using “reason” to deduce what is reasonable. Courts use the two as different “sources”. notably for juries. See, e.g., U.S. v. Kerr, 935 F.2d 276 (9th Cir. 1991) (“[A] reasonable doubt is a doubt based upon reason and common sense.”) [emphasis added].

18 A simple example should illustrate the point, namely the increasing use of AI to locate materials needed to prepare a legal opinion (Pike 2018; Goodman 2016).

19 To take a simple example, 2020 lawyering is certainly much more data-driven than 20 or 30 years ago, ands AI plays an increasingly important role in that context See e.g., Matthew Hutson, Artificial Intelligence Prevails at Predicting Supreme Court Decisions, Sci. Mag. (May 2, 2017).

20 To take a simple example, there might be a federal statute relevant to the application of a state statute in the hierarchy of the US legal system.

21 Take just this well-known example: Carlsbad Technology, Inc. v. HIF Bio, Inc, 556 U.S. 635 (2008). Looking at the statute involved in that case (28 U. S. C. §1441(c)), would lead to an entirely incorrect understanding of “the law” because the Supreme Court’s interpretation of the statute—the exact opposite of what the text of the statute actually says—is what courts are bound to follow under stare decisis.

22 How about Kant’s categorical imperative? (Kant 1998, at 25).

23 Preventing such excesses is precisely why we have many forms of regulation (O'Brien 2019; Lin, 2019, at 551).

24 To take a simple example: If I “order” my autonomous vehicle to get me to the office “as fast as possible”, does that mean it can exceed the speed limit or decide which red lights it is “safe” to run, or will its code prevent it from making those decisions? If the car misbehaves (because my instructions did not actually say to break the law), will there be a kill switch for me to use?

25 In criminal law, it gets more complicated as one wonders how a court would find that a machine has the necessary *mens rea* (Lima 2018).

26 One example among many is HAL, the computer in 2001: A Space Odyssey, that humans desperately try to shut down. Interestingly, in those books and movies, when the kill switch is triggered, it inevitably fails.

27 An example of poor design was the idea, advanced in federal legislation, of a kill switch to disconnect the United States from the rest of the Internet. See Bambauer 2011, 611.

28 In other areas, the lack of a kill switch has already been considered a source of liability. See Nixon 1998, 251.

29 For the sake of this example, assume that the choices were made autonomously by the machine. See Mcnamee 2019; Kalsnes and Larsson 2019; and Sumpter 2018.

30 https://www.appliedinvention.com/#about. See also Heckman 2019.

31 An AI machine may one day read this article in a fraction of a second and understand the discussion about kill switches.

32 Though machines will be “owned”, the legal status of the owner is not what will affect a machine’s behavior—at least not directly.

33 Naturally, a machine could theoretically be programmed in such a way that, once informed of a court order, it would “follow” that order instead of its original programming, assuming it can understand the order. However, as trying to implement this in code, complexities quickly boggle the mind (which courts, in which states or countries should it follow, to name just one issue that seems close to impossible to code). A machine could use AI to “learn” human laws but how? To take a simple example, an autonomous vehicle will likely be hard programmed to follow speed limits. If it were to learn by observation, it would likely drive above posted speed limits, as so many Americans do (Elliott et al. 2003, 964).

34 The idea that computer code can be considered a “law”, not just for machines but to a large extent also for humans, is not new of course (Lessig, 2006 and Grimmelman 2005, 1722).

35 Directly or via obligations imposed on a legal person such as their employer.

36 But what will that efficiency really cost in terms of sustaining other important social, political, economic, and environmental values?”) This can also be applied to Facebook’s AI that aims to maximize polarization and reduce our ability to have civil political disagreements.

37 This is usually referred to as putting humans on (or over) the loop (Guiora, 2017, 397; Crootof, 2016, 54).

38 Part of the literature refers to this as programming “morality”. This article uses ethics instead. As this article sees it, this distinction is crucial here because “moral” implies a determination of what is “good”, while ethics is best viewed as a set of rules that defines allowable or preferred actions. This is not a new distinction (Arnold and Scheutz 2018, 60).

39 A matter to which the article returns below. See the next Section. That said, a code of ethics need not be a top-down set of hard and fast rules embedded in AI code. A machine can use a bottom-up approach instead because AI machines can learn by constant observation and trial and error, a process described as “quest ethics”. (UK Information Commissioner’s Office and Alan Turing Institute 2020; Wallach and Allen 2009, 109–110).

40 Many examples of machine bias resulting for use of historical and partial datasets have emerged. See Paul 2019; Metz 2019.

41 This could be implemented by entrusting a specialized agency with this task, as is already the case with autonomous vehicles (Tutt 2017; Walz and Firth-Butterfield 2019, 231).

42 Such a right can be found in article 22 of the European Union’s General Data Protection Regulation (GDPR), which provides in part that a human “shall have the right not to be subject to a decision based solely on automated processing, including profiling, which produces legal effects concerning him or her or similarly significantly affects him or her.” Regulation 2016/679. See also Casey et al. 2019.

43 There would be several issues to unpack for a full analysis, including identifying the actual source of the AI, finding a court with jurisdiction and the power to issue executable orders and remedies, and dealing with sovereign immunity (Trammell and Bambauer 2015, 1159).

44 Courts may end up using the normative guardrails built into the notion of proximate cause, for example, to decide close cases. As United States Chief Justice Roberts noted in his dissent in CSX Transp., Inc. v. McBride, 564 U.S. 685 (2011), the notion of proximate cause. [S]upplies the vocabulary for answering such questions. It is useful to ask whether the injury that resulted was within the scope of the risk created by the defendant's negligent act; whether the injury was a natural or probable consequence of the negligence; whether there was a superseding or intervening cause; whether the negligence was anything more than an antecedent event without which the harm would not have occurred.” CSX Transp., 564 U.S., at 719. See also Kowert 2017.

45 In proposing those principles, the article is aware that our relationship with AI machines and their regulation (by human law) will continue to evolve.

46 The term “microdirective” is taken from Casey and Niblett 2017, 1410–1415. An additional consideration is, as this article has suggested already, that machines inform our thinking in making decisions about the data we are provided and in making recommendations. The logical next step might even be to design ethics bots that would define the proper ethical rules for us (to then try to “impose’ on them by requiring that programmers embed them in AI code. For such a proposal, see Etzioni and Etzioni 2016, 153.

47 For example, in the area of AI-based automated facial recognition, rules proposed in January 2021 by a committee of the Council of Europe suggested that “independent ethics advisory boards” be set up in each country (Council of Europe, 2021). A good example is provided by the “efforts” lead by the IEEE, self-described as “the world’s largest technical professional organization dedicated to advancing technology for the benefit of humanity.” See IEEE, 2020.

48 Those are examples taken from the code proposed by a technology company (Atlas Inc., Oct. 2019) online: https://drive.google.com/file/d/17Ys0jvxuFJi8iw_REefp0XNBqywKw4Av/view.

49 This is the case, for example, in the Ethics Guidelines for Trustworthy Artificial Intelligence posted on April 8, 2019, by the European Union High-Level Expert Group on AI, which includes both a requirement “ensuring a fall back plan in case something goes wrong, as well as being accurate, reliable and reproducible”, and that “the data, system and AI business models should be transparent.” Online: https://ec.europa.eu/digital-single-market/en/news/ethics-guidelines-trustworthy-ai (last accessed Dec. 15, 2020).

50 A useful precedent is the inclusion of labor and environmental standards in trade agreements. See Meyer, 2018, 493–94.

51 The OECD proposed not a code of ethics per se but rather a principles-based set of recommendations. (OECD 2019).

52 The term “pluralism” has many definitions. Here, the article borrows Krisch’s approach (Krisch 2012, 23).

53 The Secretary General of the UN is the depositary of “more than 560 multilateral treaties”. United Nations, Treaty Collection, online: https://treaties.un.org/.

54 A number of trade “deals” entered into recently trade a legal requirement of transparency of AI illegal. See e.g., the Agreement between the United States of America, the United Mexican States, and Canada (July 1, 2020), art 19.16(1) of the which prevents a Party (that is, Canada, Mexico and the United Sates) from requiring “access to, a source code of software owned by a person of another Party, or to an algorithm expressed in that source code, as a condition for the import, distribution, sale or use of that software, or of products containing that software.”

55 An example is the temporary IP waiver being discussed (as of early 2021) in response to COVID-19 (World Trade Organization, 2020a, b). Naturally the 2001 Declaration on the Agreement and Public Health could also be mentioned here (World Trade Organization, 2001).

56 The WTO Director-General, Nigerian Ngozi Okonjo-Iweala was appointed on 16 Feb 2021. The first African and the first woman appointed as head of the WTO, she is a development economist by training and said her priority would be to make trade work for people and for example, seeing how trade rules, including intellectual property can be tweaked to maximize access to the COVID-19 vaccines.

57 For example, the Institute of Electrical and Electronics Engineers (IEEE), published a suggestion that AI should prioritize human well-being (IEEE, 2016).

58 One example is the International Organization for Standardization (ISO 2020) In 2021, a group of multinational corporations operating in high-tech were clamoring for coördination of transnational solutions but proposing that only recommendations be developed, not binding regulatory instruments (Venkataramakrishnan 2021).

59 This observatory role could be entrusted to a new intergovernmental agency.

60 The term “specific” is used here as the adjectival form of “species”.

61 Naturally, AI machines, if they become sentient, could be protected from certain harms (from humans or from tools or entities owned or controlled by humans), a debate which other scholars have already explored (Coeckelbergh, 2010).

62 The use of “fuzzy” in relation to the legal system is borrowed from Chavkin, 1997.

63 In a separate article, we will investigate whether the law requires human control and if not, what that might mean for corporate liability for decisions made by AI machines.
